# Hospital and Institutional News

**Published:** 1918-12-21

**Authors:** 


### HOSPITAL AND INSTITUTIONAL NEWS.

### HOSPITAL EXTENSION SCHEMES IN LONDON.

Two of the rebuilding schemes in London which it is hoped to start shortly are that of the extension of the Miller General Hospital, Greenwich, and that of the New Hospital, sanctioned by King Edward's Hospital Fund, at Willesden, on a site adjoining the present cottage hospital. According to report, each scheme claims £100,000, which seems a large sum in the latter case. The proposed new hospital at Willesden will be called "The Willesden General Hospital War Memorial."

### A MUNICIPAL HOSPITAL FOR PORTSMOUTH.

WE have often regretfully recorded the financial difficulties of the Royal Portsmouth Hospital, and with equal regret the failure to remedy them notwithstanding some attempts to arrive at a policy. The culmination appears to be the recent suggestion of Dr. John Mulvany, who urges, as a council of despair, that a municipal hospital should be established because of the "limited number of bed" at the Royal and its "small resources." Dr. Mulvany rejoices therefore that Dr. Fraser and the Health Committee at last have received the sanction of the Local Government Board to build and equip a maternity hospital. What does the committee of the Royal Portsmouth Hospital say to this?

### HOSPITAL PRESENTATIONS.

Two of the first members of the committee of the Bradford Hospital and Convalescent Fund, Mr. J. B. Moorhouse, and Mr. A. T. Parkinson, have been presented with illuminated certificates of life membership. Mr. Moorhouse was the first chairman of the finance committee, and it is now ten years since Mr. Parkinson presented a trophy for the billiard tournament, through which event over £600 has been raised. The present chairman, Mr. Herbert Gill, was with Mr. Parkinson the first co-honorary secretary, and the articles of association were drafted by Mr. Parkinson. A presentation was also made to Mr. Gill.

### LORD MAYORS AND THE SURGICAL AID SOCIETY.

A FINE record of long service is possessed by the officials of the Royal Surgical Aid Society. The present Lord Mayor of London, Sir Horace B. Marshall, whose father was vice-president of the Society for fifteen years and has now been elected to that office, has served thirty-one years on the committee. The treasurer and chairman of the committee, after sixteen years' service in the latter post, has been a member of the committee since 1869, and he, Mr. Samuel Watson, has received a special vote of thanks for this continued and unfailing support. Indeed, in connection with the Royal Surgical Aid Society, the family record of Watson family is unique. The late Mr. W. H. Watson was one of the seven founders of the Society and its first treasurer, and he was succeeded by his son, Mr. Samuel Watson, the present treasurer, in 1868, on the death of his father. Mr. Samuel Watson is now an octogenarian, and the family tradition will be carried on by his son, Mr. Collier Watson, who has been a member of the committee since 1914.

### MEMORIES OF HANDEL AND JENNY LIND.

ONE of the few hospitals with the makings of an artistic tradition is the Mercer's Hospital, Dublin. Founded in 1734 by Mary Mercer, a physician's daughter, the hospital is nearly 200 years old. In 1741, Handel, who is associated in most men's minds with the Foundling Hospital only, conducted in Dublin a performance of "The Messiah" for the benefit of the institution, and when the master's centenary celebration was held in 1859, Jenny Lind sang on its behalf, and was the means of raising a large sum for the hospital. Past means of collecting money, to be remembered to-day when the need is great, included charity sermons and even a lottery, which was less frowned upon by the hard-headed men of the eighteenth century than it is by the democracy of to-day. The Lord Chancellor of Ireland, who addressed a recent meeting of Dublin citizens on its behalf, emphasised these points in its history, and we hope that the committee entrusted with the task of paying the overdraft and increasing the inadequate income will be well supported, so that an institution with such traditions may not be left in the cold after such a long record of good work.

### A FAMOUS HOSPITAL FOUNDER.

THOUGH not a hospital for the sick, the Foundling Hospital is one of the most remarkable institutions of London; its association with the brush of Hogarth and the music of Handel, and its place in literature help to explain why it is probably the best known of English charities to foreigners. The Rev. H. B. Compton has therefore done well to attempt a life of its founder, Thomas Coram, whose travels, colonial enterprise, religious enthusiasm, and tragic reward for carrying out his pet scheme for the establishment of the Foundling Hospital, contributed to a remarkable life, which ended at the age of eighty-three in relative poverty but real dignity. He secured the charter for his foundation in 1739, but his colleagues joined issue with him and succeeded in excluding the old man from all control. The subject of the work then is the difficulties which beset a man of public spirit in every age, and shows that only by a faith which is indeed lively can such men push their schemes through the jealousy and the obstruction of the average men around them. We hope for an opportunity to review the work later.

### BART.'S AND ITS OLD PRIORY CHURCH

LOVERS of St. Bartholomew's Hospital and its ancient priory church, where lie the remains of Rahere, the founder of the hospital 800 years ago, have now an opportunity of showing their appreciation of the oldest of our London hospitals. Four years hence—to be correct, in March 1923—the 800th anniversary of the founding of the Church of St. Bartholomew-the-Great will be celebrated. There is no more perfect and beautiful specimen of Norman architecture in London, and, despite the ravages of time, it still remains a silent witness of Rahere, and his work for the poor and needy of London. An opportunity has occurred of acquiring for £2,000 the last six bays of the east walk of the cloister, and it is proposed to raise funds to purchase these in commemoration of this notable anniversary. For over thirty years the work of restoration has been going on, and the latest, when completed, will see the whole of the old Priory Church available for public worship. No more fitting memorial to Rahere could be found than to restore his church to the state in which he left it. His lovely tomb was one of the first treasures in London that was protected from damage by air raids, and, now that D.O.R.A. is dying, and it is hoped will soon expire, there is no harm in saying how perilously near the church and the hospital were to destruction in those days when Zeppelins and enemy aircraft flew over the City. In St. Bartholomew's Close, near by, considerable damage was done, and some lives were lost, the hospital itself being damaged, but providentially the hospital and the church escaped.

### THE STABLE AND THE CLOISTERS.

The rector of the Church of St. Bartholomew-the-Great, the Rev. W. F. G. Sandwith, in an appeal for assistance which he has issued, states that the portion it is proposed to purchase, though now a stable, fitted with stalls, is known to contain: "An ancient entrance doorway to the Chapter House, with the usual window openings on each side; the arched entrance to the dorter or dormitory of the monastery, the east side of which was uncovered in the adjoining premises in 1903; and there is good reason to believe the entrance to the slype (or passage between the south transept and the Chapter House) still exists below the present ground level." It is to be hoped that the Rector will receive quickly sufficient funds to purchase this interesting relic of the famous church, and that when the 800th anniversary of the foundation is celebrated it will have been restored and beautified.

### SLEEPING IN THE LAW COURTS.

To slumber during the sitting of a court of law is no new thing, even, it is said, upon the bench, but a precedent has been created by the adaptation of the Great Hall into a dormitory for the crowd of American sailors now on leave from the Grand Fleet. It is the American Red Cross which has accomplished the change, and installed the great canteen which stretches across the width of the building. The occasion arose from the fear that the American sailors might have to return home without seeing London, and the Lord Chancellor thereupon granted the request of the Red Cross authorities. The capacity of the Great Hall, its alcoves and corridors, is such as to offer almost unlimited accommodation, and the "American Red Cross Bulletin," naturally appreciative of figures, records with delight that the Hall is 240 feet long and 80 feet high. The existing buffet, which serves many counsel, witnesses, and spectators during the day, was already prepared, but to allow the day's legal business to proceed everything has to be cleared away by 9 A.M. Although 1,200 men sleep in the Hall at night, the Red Cross has fulfilled its obligation in the matter, and the plan has given additional pleasure to the American sailor who likes to do the unusual thing.

### A CHAIRMAN ON THE EPIDEMIC.

A heavy toll of influenza cases has occurred in connection with the York County Hospital, where at one time 20 of the nurses out of fifty, three ward maids, and five out of eight men servants have been ill, and in two cases the illness was fatal. Unfortunately the death roll includes Miss Stewart, the late matron, who just before her death from pneumonia had been promoted to the important post of matron of the Welsh Military Hospital, Netley. The illness of the staff led to the closing of two wards, and Mr. W. F. H. Thomson lately declared that the epidemic was the worst which he had ever known. The high mortality of the cases brought to the hospital is explained by the fact that only grave cases could be admitted at all. In fact, in Mr. Thomson's view, such an epidemic as that of the present autumn was not a matter to be dealt with by a voluntary county hospital. It is curious that influenza, notwithstanding its virulence, has not been promoted to the rank of "an infectious disease" in the public mind, and in consequence its burden has been illogically thrust upon the general hospitals.

### DEMOBILISATION AND MENTAL DEFECTIVES.

A conference of local authorities held at Leeds has passed the following resolutions:—That the problem of the mentally deficient is so important that it must be dealt with even in war time; that grave danger arises from the uncontrolled lives of defective girls and women, men and boys of immoral or criminal tendencies, and that this danger will be greatly aggravated during demobilisation. The Conference urge the Government, therefore, to make the vigorous administration of the Mental Deficiency Act part of the work of Social Reconstruction, and to remove the financial limitations which prevent further action by local authorities. Miss Fox, the Secretary of the Central Association for the Mentally Defective, stated that it was intended to apply for an amending Act providing that the Education Authority should be empowered to notify low-grade children not in special schools and making additions to the reasons for notification in the case of children leaving special schools where supervision was thought desirable.

### THE DRAWBACKS OF THE VOLUNTARY HOSPITAL.

In a recent manifesto Colonel Bruce Vaughan contrasted the voluntary with the involuntary (that is to say, the municipal) hospital as follows: "The rate-aided hospital is more than counterbalanced by the moral value of the voluntary system, for it is certainly more efficient and certainly more economical, but there are certain drawbacks. The generous pay for the selfish; the conscientious for the unconscientious. This unfairness acts prejudicially, and is causing a strong feeling that hospitals should be rate-supported. The voluntary hospitals in the kingdom depend for the most part for their upkeep upon comparatively few subscribers, who appear again and again in the various hospital lists. This is so in Cardiff, and our generous donors can be counted on the fingers of our hands. But if the hospitals were transferred to a public body the taxpayer would have to pay in the near future at least one shilling in the £, and limited companies would realise that by not contributing their proper quota to the voluntary hospitals they were not doing their duty to their shareholders; but, on the other hand, they were adding a compulsory charge on their property, both lands and houses and investments." Will business men listen to business proposals, or, if charity leaves them unmoved, will not laxity yield to principles of commercial prudence?

### TEXTILES FOR METROPOLITAN INSTITUTIONS.

The Local Government Board has considered the question of the large stocks of surplus textiles in the possession of the Bermondsey Board of Guardians, bought by their clerk, Mr. E. Pitts Fenton, which were recently the subject of a lengthy inquiry by their inspector, Mr. J. S. Oxley. It appears to the Board that all stocks beyond those necessary to maintain supplies to Lady Day 1920 should be regarded as surplus stock. When this was ascertained the surplus supplies should be offered to the Metropolitan Asylums Board, but in the event of that authority not being able to make use of the goods an opportunity to purchase should be afforded to the other Metropolitan Boards of Guardians. After a deputation from the Guardians had interviewed Mr. Oxley his suggestion was adopted, that an independent gentleman should be nominated by him to consult with the chairman and chief officer of each institution to advise what stock should be disposed of.

### SPIRITS FOR INFLUENZA.

In view of representations that have been made that there is in some districts a shortage of spirits required in treatment of patients suffering from influenza, arrangements are being made by the Ministry of Food that in the event of information reaching the Ministry indicating that an additional supply of spirits is necessary in any particular district for the treatment of such patients, the Ministry will direct a special supply to that district. Steps are being taken by the Ministry to obtain representative medical opinion as to the reality and extent of the need in the various localities affected. All bottles of spirit supplied for this purpose will bear distinctive labels, and will only be sold against a certificate from the doctor attending the patient.

### THE DISCHARGE OF UNDERTAKERS.

The Local Government Board has replied to the suggestion made by the Southwark Board of Guardians, which was reported in The Hospital on December 7, that men should be released from the Army, especially those on Home service, who were skilled undertakers and coffin-makers. The Board has communicated with the Ministry of Labour with regard to skilled undertakers, and understands that the Ministry will consider the question of recommending the release of men whose names are put before them by Alderman J. R. Hurry, the honorary secretary of the Undertakers' Association. It is still so difficult to secure prompt burials in many parts of London that it is somewhat surprising to find how little the authorities have done to relieve the situation. The delay to a great extent is due to the number of Government departments dealing with the same subjects and problems, with the inevitable result that there is a considerable amount of overlapping and delay.

### OFFICIAL SCALE FOR THE WAR BONUSES.

The Local Government Board has forwarded to the various Guardians the scale of war bonuses now authorised for permanent Government officials in the Civil Service, in accordance with recent awards of the Conciliation and Arbitration Board for Government Employees. The Board has been willing to authorise additional remuneration for the period of the war to officers whose remuneration required their sanction, within but not beyond the scale authorised for Civil Servants. Such payments, therefore, are within the discretion of the Guardians. The Board recognised that, though local conditions vary, the Guardians might properly take the Civil Service scale for a guide in dealing with non-resident whole-time officers. The case of resident officers depends on the provision, in whole or in part, of rations at the cost of the Guardians. The allowance of part-time officers would doubtless be regulated by their special circumstances. The adoption of the Civil Service scale may remove some of the anomalies that have arisen through the piecemeal fashion adopted by public authorities in dealing with war bonuses. Some officers in the lower grades of employment have been receiving with their war bonuses more salary than those above them. The Civil Service scale appeared on page vi of The Hospital of December 14.

### NEW FARM COLONY FOR CONSUMPTIVES.

Dr. J. Wright Mason, the Hull medical officer of health, reports progress with a scheme for the after-care of patients who have undergone sanatorium treatment. Each case is dealt with entirely on its merits. It is recognised that when a man has completed a course of treatment in an institution he is not always fit to resume his ordinary occupation. He is, therefore, when necessary, advised to undergo a short period of convalescence, during which time assistance in the form of extra nourishment is granted. The After-Care Committee, through the munificence of Sir James Rickett, has been able to acquire an estate at Walkington to be used as a farm colony for the benefit of convalescent consumptive patients. It is intended that the work of the colony shall principally consist of market gardening, horticulture, poultry-keeping, and pig-rearing, whilst later on, when more land is acquired, it may be possible to extend the occupation to agricultural pursuits. Only arrested cases ,selected by the medical officer are sent to the colony—that is, persons who have previously undergone treatment in a sanatorium.

### A TYPE OF A GROUP OF INSTITUTIONS.

Homes for advanced cases, except for certain diseases, are relatively rare, but they form a distinct group among institutions. The nature of their work demands a cheerful setting, and since cheerfulness is the sign manual (so to speak) of the religious life, a conventual note is characteristic. Such a note naturally associates itself also with a denominational atmosphere, and these homes are usually connected, if not with sisterhoods or the like, at least with one of the branches of church or chapel. The administrative atmosphere of these homes is to some extent peculiar. A case in point is St. Columba's Hospital, or Home of Peace, in Avenue Road, Swiss Cottage. Known till lately as "Friedenheim," Miss Davidson's foundation, more than once enlarged, is a home for the dying. The late Dr. Habershon, a physician of distinction, was an active member of its council; and among the clergy associated with it is Prebendary Webb-Peploe. The place is made cheery with flowers, which are to be seen always and everywhere, and "a special mode of conveying the Gospel message is by means of texts painted on the walls." Perhaps, amongst others, the home's chief claim to merit is that no voting is necessary to secure admission. Nearly all wards are free, and they include an Annie Zunz ward for women suffering from cancer, and a children's ward also. The mortuary is probably unique in its design and furniture. The latter includes three iron bedsteads, resembling those used in the wards, which are covered by a violet pall, fringed with a white border which is embroidered with a text. The chaplain, who is still, we believe, the Rev. H. E. Noyes, is probably busier than other institutional chaplains. No case—and all are necessarily in the chronic or later stage—is allowed to die without the consolations of the Church. An account of St. Columba's was written by Mr. L. A. Streetly-Smith three years ago, and this contains a photograph of the mortuary. The new secretary to the hospital is Mr. Arthur R. Godrich.

### DRUG TAKING IN THE WEST END.

Great interest has been aroused by the disclosures, made at the inquest on a young actress, of the habit of "doping" and drugging in certain circles of people in the West End of London. The Sunday papers have had an outburst of "pseudo-hysteria" (to quote the *Pharmaceutical Journal*) on the subject, and although the reports are generally exaggerated, there seems to be evidence of a certain amount of traffic in narcotic drugs. As regards cocaine and opium (solid), very stringent regulations under the Defence of the Realm Act require that these may only be supplied on the prescription of a registered medical practitioner, who must sign his full name, address, and the words "not to be repeated." It is only fair to pharmacists as a body to state that they observe the regulations laid down and rigidly adhere to the law governing the supply of poisonous substances. In fact, many of the general public, and even some medical practitioners, regard pharmacists as being too punctilious in the observance of the regulations. In one case last week an inquest story revealed the fact that a West End firm of chemists had refused to supply veronal without a prescription, though unfortunately the subject, a V.A.D. nurse, had access to other drugs, so that the refusal of supplying veronal did not prevent loss of life in this particular case.

### THE FORGING OF PRESCRIPTIONS.

One great problem is the forging of prescriptions, which is not always easy to detect. It might be a good plan if, in every case, the pharmacist who dispenses a prescription of cocaine and other narcotics notified the medical man concerned that a prescription had been dispensed, giving date and other particulars. How cocaine and opium get into the hands of the illicit traffickers is difficult to imagine, but no doubt the police officials will follow the matter and endeavour to trace the source of first supply. Institutional medical officers frequently have "drug-takers" under treatment, and they could greatly assist the authorities by attempting to ascertain where these victims have obtained the substances.

### THIS WEEK'S DRUG MARKET.

The near approach of the Christmas holidays, and the end-of-the-year stock-taking period, have taken away what little life was left in the drug market. With the exception of the continued steady demand for drugs for which there is a ready public sale—as, for instance, eucalyptus oil—there is very little inquiry and still less business. When things become active again, as they should early in the New Year, it will be interesting to see if, and how far, prices have receded in the meantime. Cascara sagrada is in better supply, and the price is lower and likely to be lower still. Gum acacia, senega root, myrrh, juniper berries, scammony roots, sarsaparilla have a downward price tendency. Buyers of bromides are holding aloof, for although supplies are practically controlled at an official price, it is anticipated that when the control is removed the market will follow the lead of the market in the United States, where prices have dropped substantially. Values of several of the synthetic drugs—*e.g.*, amidopyrin, phenazene, sulphonal and aspirin—are well maintained, but on the other hand hexamine, paraldehyde, phenacetin, and salicylic acid have a downward tendency. Sugar of milk is again lower, and is now once more quoted by the hundredweight instead of by the pound.

